# The correlation between sperm DNA methylation and DNA damage: a comparison of comet and TUNEL

**DOI:** 10.3389/frph.2025.1523386

**Published:** 2025-02-20

**Authors:** Hailey Zimmerman, Tim Jenkins

**Affiliations:** Department of Cell Biology and Physiology, Brigham Young University, Provo, UT, United States

**Keywords:** DNA methylation, comet, TUNEL, DNA damage, sperm DNA, comet assay, TUNEL assay

## Abstract

**Objective:**

To evaluate the relationship between sperm DNA methylation and DNA damage as determined by the comet or TUNEL assays.

**Design:**

Retrospective research study.

**Setting:**

University-based andrology and *in vitro* fertilization (IVF) laboratory.

**Patient(s):**

Data came from 1,470 male partners (ages 18 and older) recruited from heterosexual couples (ages 18–45 years) seeking fertility treatments. These data were analyzed retrospectively from the Folic Acid and Zinc Supplementation Trial (FAZST) study.

**Main outcome measure(s):**

Comet and TUNEL measures and associations with DNA methylation patterns.

**Result(s):**

Comet and TUNEL values were correlated with one another across all samples (*R*^2^ = 0.34, *P* < 0.001); however, when assessing the highest and lowest scores reported from each assay, there was little overlap between patients. This suggests that Comet and TUNEL are identifying key differences that may be meaningful and correlated with other sperm metrics. We compared both comet and TUNEL to methylation array data using a sliding window analysis, which identified far more significantly differentially methylated sites as a function of comet than TUNEL (3,387 vs. 23). Interestingly, sites associated with comet were associated with biological pathways related to DNA methylation involved in germline development, as determined by a GO term analysis. The TUNEL assay, by comparison, produced no relevant biological pathways.

**Conclusion(s):**

Because the comet and TUNEL assays are both used to indicate levels of DNA damage, and outputs of both are correlated to each other, it would seem to follow that both are equally predictive of deviations in DNA methylation patterns. The findings of this study suggest that this is not the case. The comet assay shows a significantly higher association with DNA methylation disruption, and we therefore believe it to be a better indicator of sperm epigenetic health.

## Introduction

DNA methylation can be a powerful predictor of tissue health. In sperm, data have suggested that DNA methylation can predict pregnancy outcomes for those with infertility, and specifically those undergoing *in vitro* fertilization (1–3). Although the mechanisms are not well understood, sperm from infertile men have been shown to display distinct DNA methylation profiles compared to fertile controls. DNA methylation is a biological process in which methyl groups are added to the fifth carbon of the cytosine residue. Methyl groups are added most frequently at cytosine-guanine dinucleotides, also known as CpG sites. DNA methylation is an important regulator of gene expression and thus it plays a role in many cellular processes. Aberrant methylation of DNA promotes genome instability and abnormal gene expression in many human diseases and can thus act as an epigenetic biomarker for these disorders (4). When genomic regions are methylated abnormally, gene expression is often directly affected (5), which can heavily interfere with regular biological processes and prevent important cellular functions (6).

Physical DNA modifications can also occur through DNA damage, including single or double-stranded breaks. Damage can occur due to normal cellular processes or environmental exposure to damaging agents and, when unrepaired, can result in mutations or disease. DNA damage has been shown in previous research to correlate with decreased implantation rates and the rates of continuing pregnancies for those undergoing intracytoplasmic sperm injections (ICSI) treatments (7).

The comet assay and the Terminal Deoxynucleotidyl Transferase dUTP Nick End Labeling (TUNEL) assay are two distinct but common ways to quantify levels of sperm DNA damage. The comet assay, which is also known as single-cell gel electrophoresis, measures nucleic acid damage as a cell migrates under an electric current in a gel. Normal DNA remains in a head-like shape while the tail of fragmented damaged DNA stretches outward (8). The result resembles a comet shape, which inspired the assay's name. Comet results have been considered the most sensitive method of recognizing double-stranded breaks (9). The TUNEL assay detects DNA damage fluorescently using an enzyme called dUTP (10). Results from this assay produce a DNA fragmentation index, called a DFI score.

Interestingly, tumor cell DNA damage has appeared to correlate with global DNA methylation pattern abnormalities (11). Further research specifically identified unique DNA methylation signatures associated with sperm DNA damage (12). These findings suggest that high levels of DNA damage are associated with significant changes in DNA methylation, and that DNA damage may be affecting methylation patterns in human sperm. We wanted to assess this relationship between sperm DNA damage (as predicted by both the comet and TUNEL assay) and regional DNA methylation patterns in sperm specifically.

In addition to identifying methylome disruptions tied to damage, we also aimed to evaluate the two DNA damage assays. The comet and TUNEL assay are used commonly and somewhat interchangeably within reproductive research. In studies comparing multiple sperm DNA fragmentation tests, the alkaline comet assay shows to be the best predictor and most consistent, with the TUNEL assay trailing right behind (13, 14). However, little to no work has been done directly comparing the two common assays. Since they are both assumed to be efficient measures of DNA damage, it is reasonable that DNA damage measured by each should correlate with each other and similar deviations in DNA methylation patterns. Therefore, we compared the results between these two common DNA damage assays.

## Methods

### Data

We obtained Infinium EPIC methylation array data from 1,470 sperm samples in the previously published Folic Acid and Zinc Supplementation Trial (FAZST) study via a data use agreement (DUA) (15). The EPIC array can detect cytosine methylation at over 850,000 CpG sites in the global genome (16). Raw methylation values were normalized using the minfi package in R and SWAN normalization resulting in the generation of beta values. Beta values range from zero to one and represent the percentage of methylation at a single CpG site. These scores allow an intuitive interpretation of epigenetic data. Each sample was also analyzed for DNA damage using both the comet and TUNEL assay as part of the FAZST study. We did not perform the TUNEL and comet assay in this analysis, and are only analyzing the data from the previous FAZST study. TUNEL and comet were performed in the FAZST trial for all samples, assays were performed by standard procedures commonly used for human sperm. As a brief summary- the comet assay utilizes single-cell gel electrophoresis, measuring nucleic acid damage as a cell migrates under an electric current in a gel. The TUNEL assay in contrast detects DNA damage fluorescently using an enzyme called dUTP, producing a DNA fragmentation index, called a DFI score. Each sample has a comet and DFI score associated with it. Samples were stratified independently by both comet and TUNEL (DFI) scores and groups were produced based on the highest and lowest 10% of scores. By studying the high and low DNA damage groups, we were able to see differences in sperm samples which had high indicated damage vs. those with little to no single or double stranded breaks.

Before running analyses, somatic cell contamination was checked. Blood, tissue, or plasma cell contamination can heavily skew sperm DNA methylation signatures (17). To accomplish this, we used a novel pipeline comparing each sample's DNA methylation signature to somatic cell DNA methylation patterns. More specifically, this pipeline evaluates fraction methylation at the DLK1 locus as a cutoff as has been done in previous studies (18). Out of 1,470 samples, 79 were evaluated to be likely contaminated with somatic cells (Supplementary Figure 1). These samples were removed before we continued with the project. We went on to compare the high comet scores group (*n* = 69) to low comet (*n* = 147), and high TUNEL score group (*n* = 90), to low DFI (*n* = 142).

### DNA damage assay comparison

First, the TUNEL and comet measures were compared to each other. The assay data were assessed using visualizations, confirming that the assumption of normality was reasonably met. This process began with a simple linear regression of comet and TUNEL scores using linear model and ggplot functions R. If the comet assay predicts higher DNA damage, the TUNEL assay may or may not do the same. A regression was performed from all patients with TUNEL and comet scores (*n* = 1,470). A *p*-value of <0.05 was considered significant and an *R*-squared measure was additionally reported to describe correlation.

### USEQ analysis

Four sample groups, composed of high DFI, low DFI, high Comet, and low Comet scores were produced to facilitate various bioinformatic analyses. We began by assessing significant differential methylation. This was done by performing a sliding window analysis (USEQ analysis) using a command line java application to aid in analyzing next generation sequencing data (19). This program runs through over 850,000 CpG sites in the genome included on the EPIC array and compares each site between a control and treatment group using a Wilcoxon Signed-Rank Test. In this case, we defined the control as those with low indicated DNA damage, and treatment as those with high indicated DNA damage.

After completion, a file is created containing genomic regions with statistically significant differences in methylation between samples of high and low damage (as indicated by either the comet or TUNEL assay). Those sites with observed differences were termed as “differentially methylated regions”, or DMRs. A phred-scaled false discovery rate (FDR) is associated with each region. We chose to extract those with an FDR score above 13 (correlating with a *p*-value of 0.05) for absolute accuracy and statistical significance when analyzing DMRs. We calculated mean beta values for each chromosomal site within each group, and then pulled out those with the highest differences in beta values. These differences were then plotted (Figures 2a–b).

### Gene ontology analysis

Using these DMRs, we sought to determine if our significant DMRs were related to any gene ontology (GO) or cellular pathways. To answer this question, we conducted a GREAT analysis using the online tool created by Stanford University (20). Notable epigenetic pathways were listed which related to DMRs between the comet and TUNEL groups. GO outcomes between the two assay groups were identified.

### Instability analysis

To further understand sperm cell abnormality through DNA methylation, we conducted an epigenetic instability analysis. This novel analysis was produced to show variability in methylation between groups (21). This program compares DNA methylation signatures at every site covered on the array to those of a healthy and fertile sperm donor cohort and marks specific sites as “unstable” or variable. Unstable sites are defined as those in which DNA methylation is disrupted as defined by Pollard et al. (22). This analysis produces both a chaos and a variance score. The chaos scores indicate how many gene promoters are unstable, with an extreme difference in methylation compared to what the average healthy sperm methylome produces. The variance score produced relays how variable the methylation signatures were in comparison to the healthy methylome. Results were visualized in violin plots which graphically display differences in stability.

### Epigenetic age

Lastly, an epigenetic age analysis was then performed using the Jenkins Germ Line Age Calculator (18). Epigenetic age describes an individual's biological age as measured by DNA methylation rather than their chronological age, which describes the length of time a person had lived. An individual's sperm cells may be biologically older than their chronological age, which may be associated with adverse pregnancy outcomes (23). We were able to identify the recorded epigenetic age of those with high DNA damage and those with indicated low DNA damage. By including this calculation, we aimed to determine if there was any correlation between sperm DNA damage and epigenetic age, and how “aged” the DNA was in each sample.

## Results

### Comet vs. TUNEL

**Figure 1:**
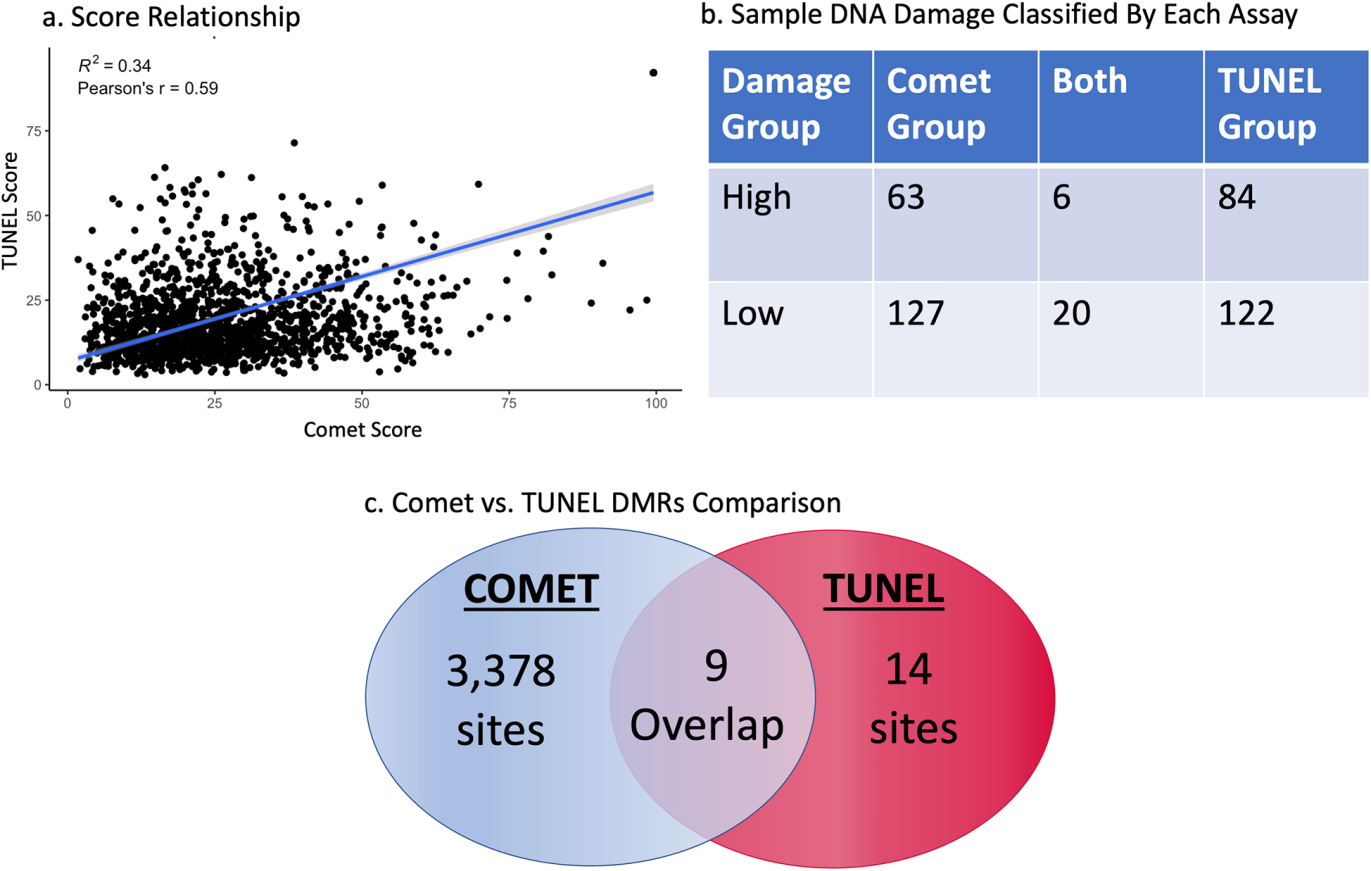
Regression of comet and TUNEL DNA damage scores from all patients (*P* < 0.001) *R*^2^ = 0.34 **(a)** the highest and lowest 10% of DNA damage samples as set by the comet and TUNEL assay, after the data was cleaned for somatic cell contamination. This table reveals how many samples were determined by both assays to have low or high DNA **(b)**. All differentially methylated sites between the comet and TUNEL assay as determined by a sliding window analysis **(c)**.

Sperm cell DNA damage scores taken from the comet and TUNEL assay revealed a positive relationship, with a *p*-value less than 0.001, Pearson's r of 0.59, and an *r*-squared of 0.34 (Figure 1a). However, each assay indicated very different sets of patient samples with low or high DNA damage. The high comet scores group contained 69 samples; the grouping of high TUNEL scores held 90 samples. Only 6 of these samples were the same between both groups. These 6 samples constitute only 4% of total individuals identified to have high DNA damage. This pattern is repeated in the low DNA damage groups, with 147 samples in the low comet group and 142 in the low TUNEL group, with 20 samples identified by both assays. This describes only 7% of samples with little to no DNA damage were classified as “low” between both assays (Figure 1b). Each assay appears to identify completely different sets of patients for indicated DNA damage levels.

### Sliding window analysis

Our first analysis provided insights into specific regions of the genome which were differentially methylated between high and low damage groups. A significant DMR was classified as holding a phred-scaled false discovery rate ≥13. The USEQ analysis reported 3,387 significantly differentially methylated regions (DMRs) between high and low comet score groups, and 23 between high and low TUNEL score groups (see Figure 1c). Only 9 chromosomal regions were found to be the same between the two groups; differentially methylated between high and low comet as well as high and low DFI. It is noteworthy that DMR's reported between the comet groups contained regions holding a phred-scaled false discovery rate ≥40, while none of the TUNEL groups significance were reported with a significance that high.

**Figure 2:**
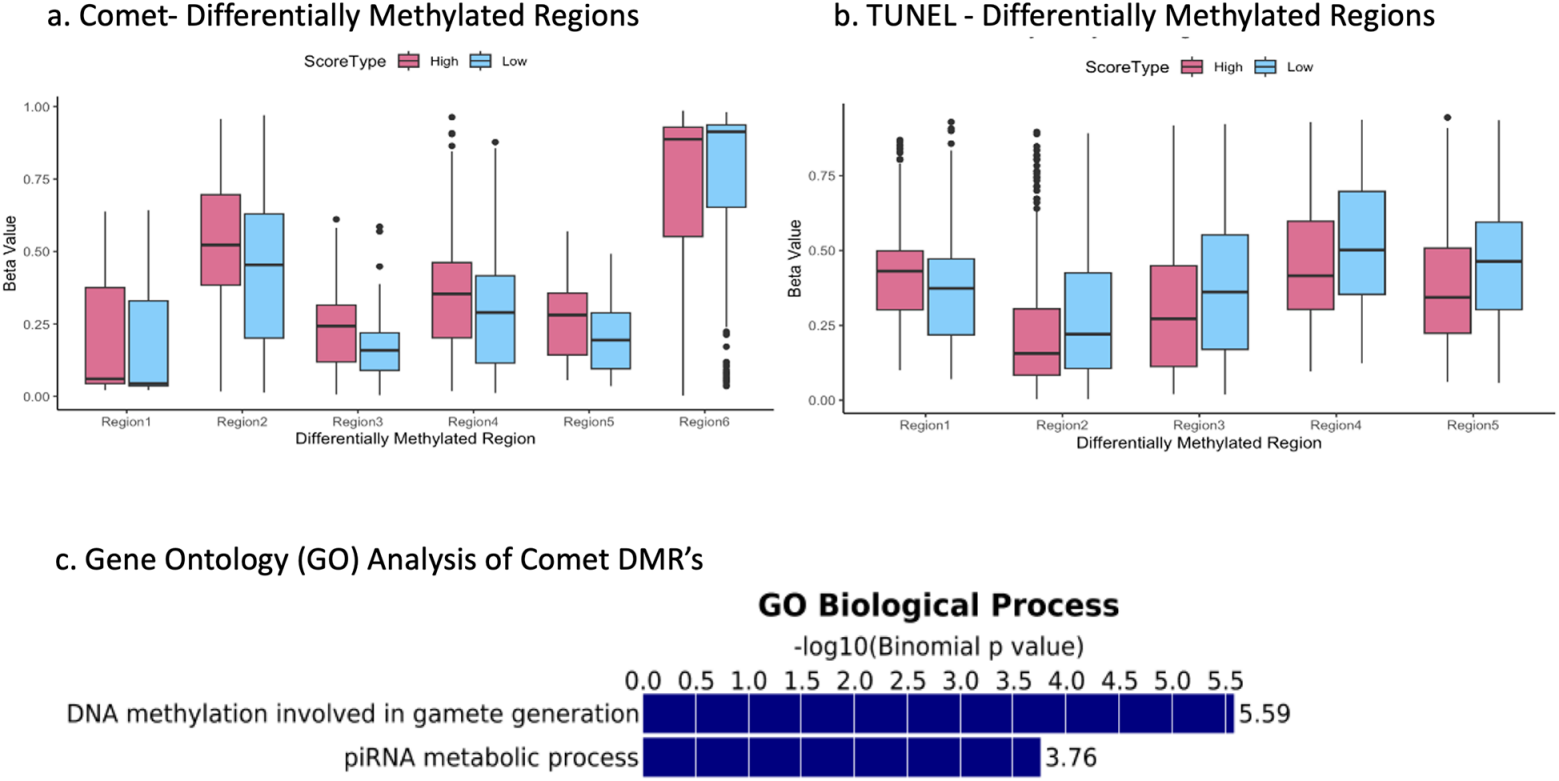
The top 6 most differentially methylated sites for the comet group **(a)** and the top 5 for the TUNEL group **(b)**- each site held a difference in beta values of 0.07 or greater. Binomial *p* values relating COMET associated differentially methylated regions to gene-ontology processes **(c)**.

Boxplots were created to illustrate differences in DMRs found between the high and low comet scores groups in the USEQ analysis. We decided to plot DMR's with a difference in beta value of 0.07 or higher (Figures 2a–b). These plots express subtle differences in methylation signature. Regional differential methylation revealed 6 significantly altered regions (5 hypermethylated; 1 hypomethylated) between high and low comet categories and 5 significantly altered regions (1 hypermethylated; 4 hypomethylated) between high and low DFI values in the TUNEL group, as determined by our threshold. These regions were located across various chromosomes. Regions for comet group were completely unique and independent from the TUNEL group, with no chromosomal regions overlapping within the regions with the greatest change in DNA methylation signature.

### GREAT analysis

Differentially methylated sites as determined by the sliding window analysis were put into a GREAT analysis to identify relevant gene ontology (GO) pathways. The 23 DMRs (as determined by a sliding window analysis) between the high and low TUNEL score groups showed no relationship to any GO pathways. However, 3,387 significant DMRs between the high and low comet score groups were highly correlated with two GO pathways associated with germline development (Figure 2c). The first was DNA methylation involved in gamete generation, and the second was involving piRNA metabolic process. This finding was the most valuable in our work. This analysis demonstrated those significant DMR's as determined by the comet assay were directly linked to DNA methylation GO pathways, while the TUNEL assay DMR's were not.

**Figure 3:**
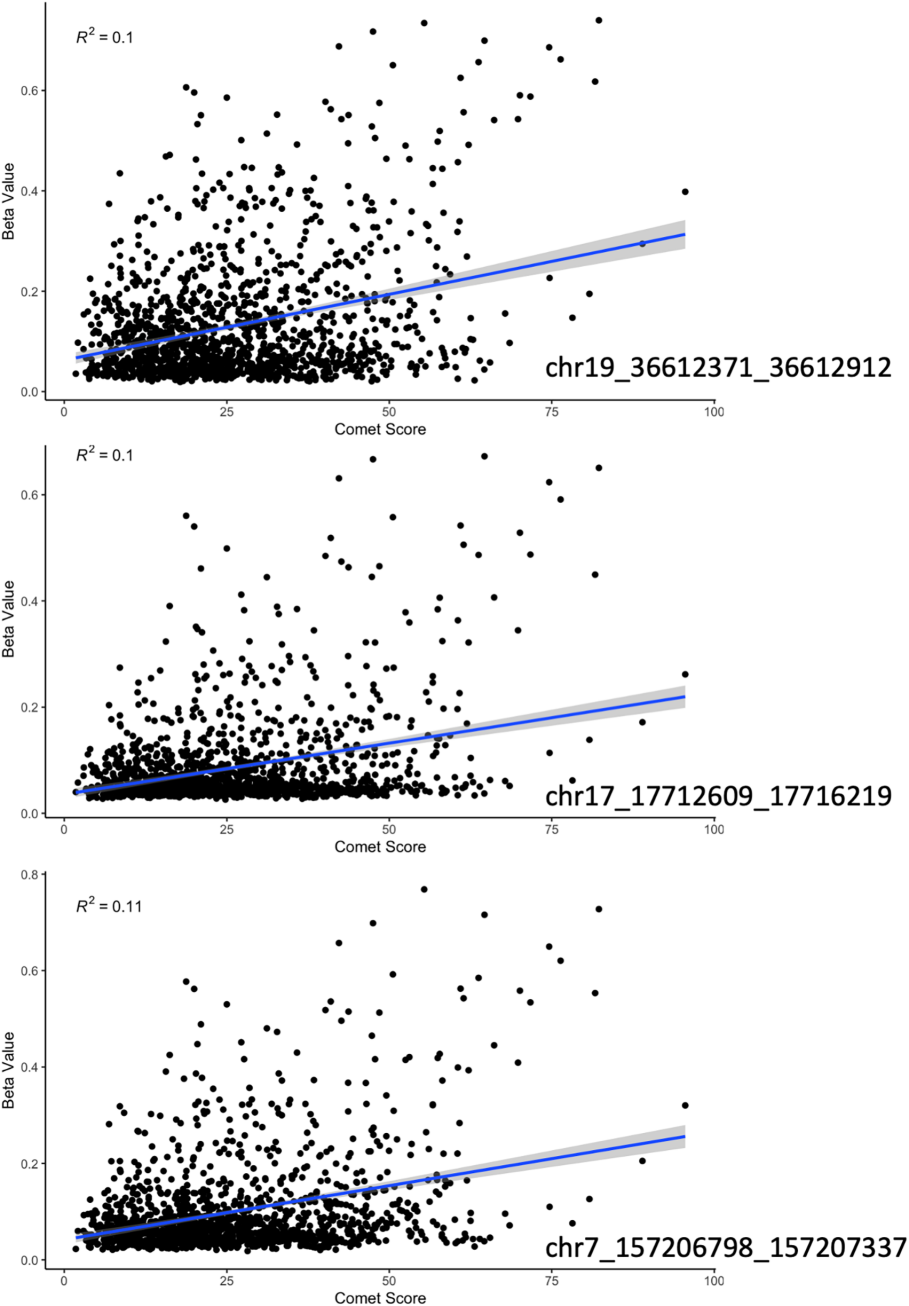
A regression of beta value with increasing damage score (as indicated by the comet assay) at the three most significant sites according to our sliding window analysis. Each region had a positive correlation with a *p*-value less than 0.01 and an *R*-squared of approximately 0.1.

We continued to dive into these results with a linear regression of comet scores and beta values at the top 3 most significant DMR's as determined by USEQ. All three depicted significant positive relationships with a *p*-value less than 0.001 (Figure 3).

### Instability analysis

**Figure 4:**
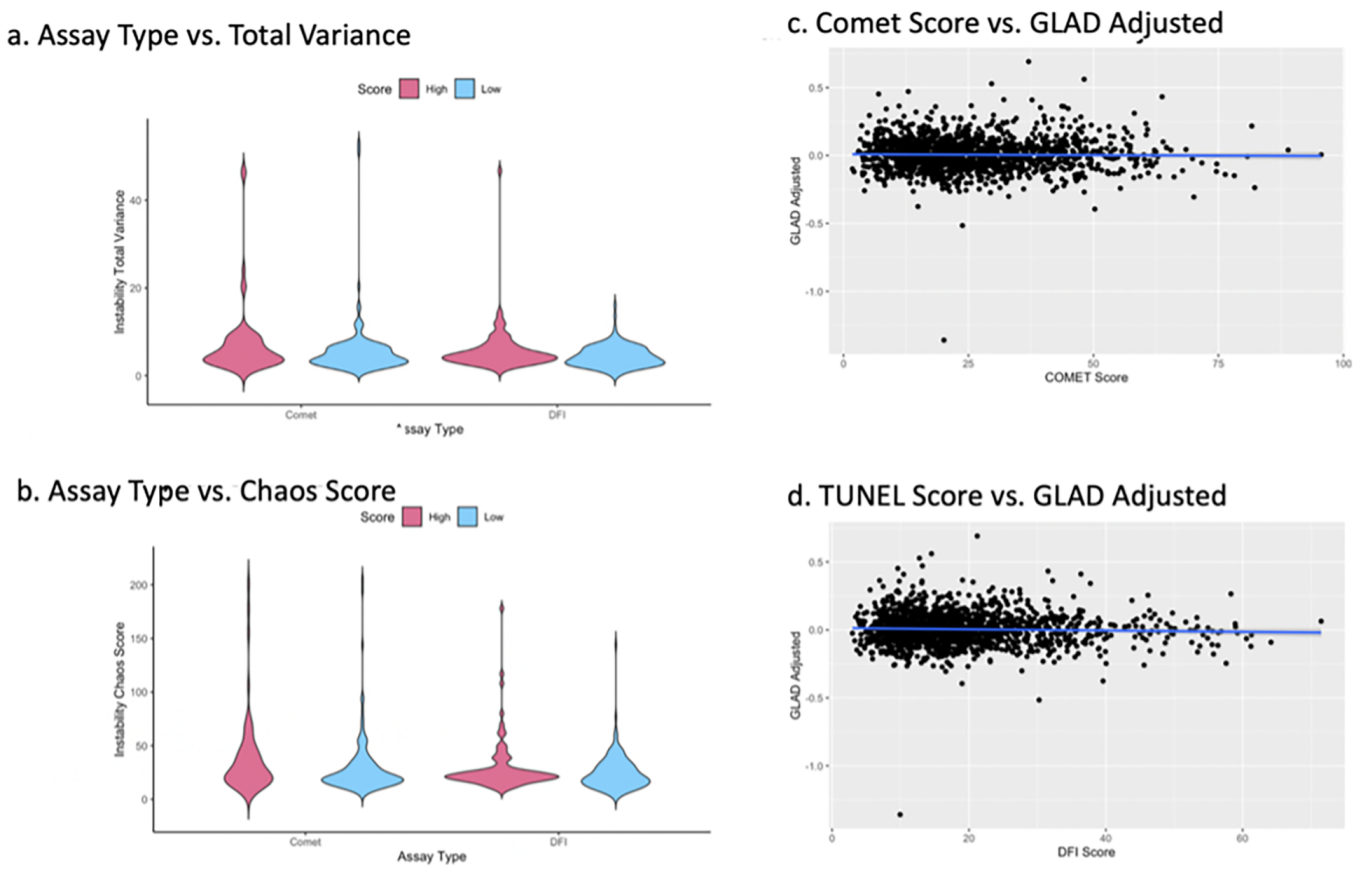
The total variance distribution in sperm DNA methylation signatures from each group in both assay as compared to a fertile sperm donor, insignificant difference between groups with *P* > 0.05 **(a)** chaos score distribution across sample groups, insignificant difference between groups with *P* > 0.05 **(b)** scatterplots depict differences in epigenetic and recorded age, compared to DNA damage score from both the comet **(c)** and TUNEL **(d)** assay.

We did not identify significant changes in instability between samples with high or low DNA damage using either assay (Figures 4a–b). Distributions of chaos and total variance scores were similar between the low and high DNA damage groups in each assay type.

### Epigenetic age

Epigenetic age was not significantly different compared to a patient's observed age between high and low Comet or high and low DFI groups (*p* > 0.05). A linear regression of DNA damage scores and Germ-line Age Differential (GLAD) adjusted within both Comet and TUNEL groups showed no correlation (Figures 4c–d). GLAD scores are calculated by the following equation [GLAD = (epigenetic age/chronological age)−1]. In brief, as DNA damage increased, deviations from a sample's biological age remained consistent. No statistically significant difference was identified.

## Discussion

Our team was able to elucidate two important findings. First, we observed DNA methylation alterations associated with DNA damage as measured by two distinct assays. Secondly, we were able to compare these epigenetic differences between Comet and TUNEL DNA damage. We acknowledge that using previously collected data limits the ability to control potentially important variables that were not measured in the original FAZST study. Additionally, the data were cleaned from somatic cell contamination before any analyses were performed. We recognize this could in theory create a bias by excluding somatic cell contamination as a variable if it were related to DNA damage. However, it was more important to isolate our variables of interest to accurately study DNA damage and methylation disruption.

Although there seems to be a positive relationship between the comet and TUNEL assay scores, they depict two different stories when examining the extremes of scores (both high and low DNA damage). As seen in the assay comparison analysis, a 4% and 7% similarity in high and low score groups indicate mostly exclusive sets of patients with variable levels of DNA damage. Each assay appears to identify completely different sets of patients for indicated DNA damage levels at the extremes. Further, our sliding window analysis demonstrated a striking difference in the number of DMRs between each DNA damage assay. The comet analysis identified 3,364 more significantly differentially methylated sites based on DNA damage than the TUNEL assay. Those sites with the greatest differences were completely different chromosomal regions according to assay type. If comet and TUNEL are utilized interchangeably to assess DNA damage, and DNA damage is highly correlated with DNA methylation, then they should both report similar deviations in DNA methylation. However, they do not. All these data point to the same observation: the comet and TUNEL assay quantify samples in unique and separate ways. This is observable at the epigenetic level, as we noticed a remarkable difference between these assays.

Though our data clearly indicate differences in score outcomes between the two DNA damage assays, the comet assay seems to be a better candidate for measuring sperm epigenetic health. Previous studies have proven DNA methylation and damage levels should be associated (11). The comet assay supports this finding, as it seems to have a much higher association with methylation disruption, while the TUNEL group did not. Most significantly in our findings, this idea is strengthened as we observe the results from our pathway analysis; a tremendous number of the comet group's DMRs were associated with germline development. Specifically, DNA methylation in gamete generation. The DMRs from the TUNEL group were not associated with any GO terms. With the difference in GO hits, this finding heavily supports the idea the comet assay can predict epigenetic patterns, while TUNEL cannot.

This work also supports the idea that DNA damage is correlated with DNA methylation based on many measures. Thousands of differentially methylated regions resulting from our USEQ analysis show high DNA damage was associated with significant regional alterations throughout the methylome. However, epigenetic age was not significantly altered based on DNA damage status. As DNA damage increases, there were no significant changes to a sample's adjusted epigenetic age. Conversely, DNA methylation was far more variable in the high damage groups than in the low damage groups.

Although the comet and TUNEL assay are both used in reproductive research to assess levels of DNA damage in sperm, this project has concluded their link to epigenetic markers showcase very different patterns. Future work should be conducted further investigating the specific colocalization between sperm cell DNA damage and methylation. We hope to explore more results identifying potential regions of interest in which there may or may not be a link between these phenomena.

## Data Availability

Publicly available data sets were analysed in this study. The data can be found here: https://www.ncbi.nlm.nih.gov/geo/query/acc.cgi?acc=GSE185920.

## Supplementary material

Supplementary Figure 1 Somatic cell contamination across all 1,470 samples. Methylation at each CpG site within the DLK1 locus is expressed in a heatmap. Samples with a mean beta value greater than 0.25 across these CpG sites were classified as likely contaminated with somatic cells and were removed from the analysis. 79 samples were likely contaminated and labeled in pink.
