# Supplementary figures and images for: The correlation between sperm DNA methylation and DNA damage: a comparison of comet and TUNEL
Source: Front Reprod Health. 2025 Feb 20;7:1523386. doi: 10.3389/frph.2025.1523386 (PMC11882583; doi:10.3389/frph.2025.1523386)

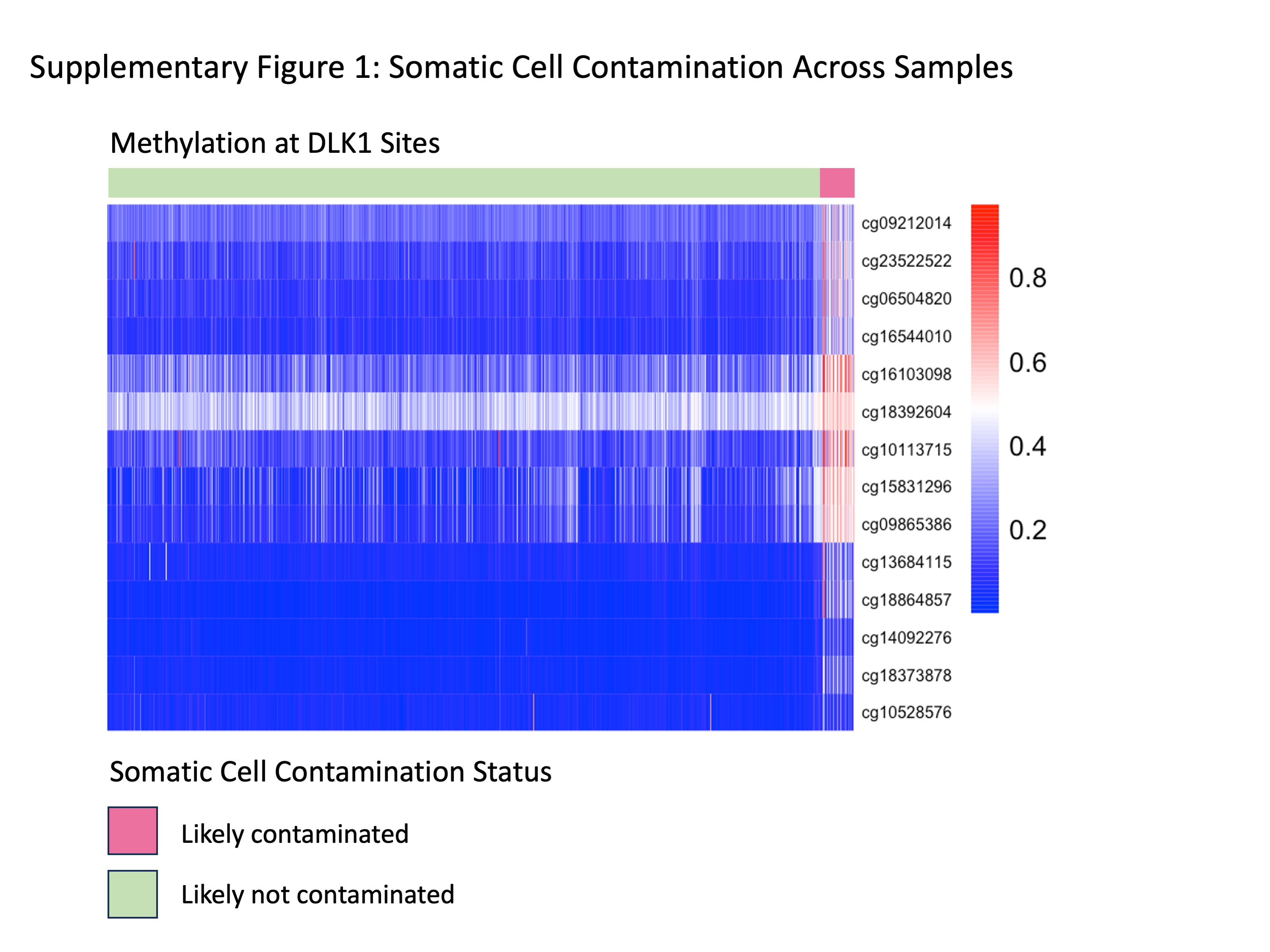

Supplement: Supplementary Figure 1 [file Image1.jpeg]
